# Physical Activity, Sleep, and Hypertension Among U.S. Middle and Older Adults: NHANES Insights

**DOI:** 10.1093/geroni/igaf122.4173

**Published:** 2025-12-31

**Authors:** Dennis Miezah, Thomas Hinneh, Rockson Ansong, Qian Song

**Affiliations:** University of Massachusetts Boston, Boston, Massachusetts, United States; Johns Hopkins University, Baltimore, Maryland, United States; Louisiana State University, Baton Rouge, Louisiana, United States; University of Massachusetts Boston, Boston, Massachusetts, United States

## Abstract

Hypertension is a primary risk factor for cardiovascular disease. Physical activity and sleep are critical determinants of blood pressure. This study investigated associations between physical activity, sleep duration, and hypertension in middle-aged and older adults. We analyzed cross-sectional data from the National Health and Nutrition Examination Survey of adults aged 40-80 years (N = 9,133). Hypertension was defined as systolic blood pressure ≥130 mmHg, diastolic blood pressure ≥80 mmHg, or current antihypertensive medication use. Physical activity was categorized as not meeting guidelines (< 500 MET-min/wk), meeting guidelines (≥500-< 1000 MET-min/wk), and exceeding guidelines (≥1000 MET-min/wk). Sleep duration was classified as short (< 7 hours), recommended (7–9 hours), or long (>9 hours). Descriptive statistical analyses were performed. Logistic regression was used to estimate the associations between lifestyle behaviors and hypertension, with adjustments made for sociodemographic and clinical factors. Overall, 61.2% of participants had hypertension. Middle-aged adults (40–64 years) comprised 69% of the sample; older adults (65–80 years) comprised 31%. Meeting physical activity guidelines was associated with lower odds of hypertension (adjusted OR = 0.78, 95% CI: 0.65–0.94, p < 0.01). Short sleep was associated with higher odds of hypertension (adjusted OR = 1.32, 95% CI: 1.12–1.55, p < 0.01). No significant association was observed for long sleep. Stratified analyses suggested stronger associations among older adults and lower-income groups. Physical activity and sleep are independently associated with hypertension in U.S. middle and older adults. Integrated lifestyle interventions may reduce hypertension burden, particularly among high-risk populations.

